# Aortic Arch Calcification Is a Strong Predictor of the Severity of Coronary Artery Disease in Patients with Acute Coronary Syndrome

**DOI:** 10.1155/2019/7659239

**Published:** 2019-08-07

**Authors:** Xiaoteng Ma, Fangjie Hou, Jing Tian, Zhen Zhou, Yue Ma, Yujing Cheng, Yu Du, Hua Shen, Bin Hu, Zhijian Wang, Yuyang Liu, Yingxin Zhao, Yujie Zhou

**Affiliations:** ^1^Department of Cardiology, 12th ward, Beijing Anzhen Hospital, Capital Medical University, Beijing Institute of Heart Lung and Blood Vessel Disease, Beijing Key Laboratory of Precision Medicine of Coronary Atherosclerotic Disease, Clinical Center for Coronary Heart Disease, Beijing 100029, China; ^2^Department of Cardiology, Qingdao Municipal Hospital, Qingdao 266000, China; ^3^Department of Nuclear Medicine, Beijing Anzhen Hospital, Capital Medical University, Beijing 100029, China; ^4^Department of Radiology, Beijing Anzhen Hospital, Capital Medical University, Beijing 100029, China

## Abstract

**Background:**

The purpose of this study was to investigate the correlation of the extent of aortic arch calcification (AAC) detectable on chest X-rays with the severity of coronary artery disease (CAD) as evaluated by the SYNTAX score (SS) in patients with acute coronary syndrome (ACS).

**Methods:**

A total of 1,418 patients (344 women; 59 ± 10 years) who underwent coronary angiography for ACS and were treated with coronary revascularization were included in the present study; chest X-rays were performed on admission. The AAC extent was divided into four grades (0–3). SS was calculated based on each patient's coronary angiographic findings. The relationship between the AAC extent and SS was assessed.

**Results:**

The AAC extent was positively correlated with SS (*ρ* = 0.639,* P* < 0.001). In the multivariate analysis, compared with grade 0, odds ratios (ORs) of AAC grades 1, 2, and 3 in predicting SS >22 were 12.95 (95% CI, 7.85–21.36), 191.76 (95% CI, 103.17–356.43), and 527.81 (95% CI, 198.24–1405.28), respectively. Receiver operating characteristic curve analysis yielded a strong predictive ability of the AAC extent for SS >22 (area under curve = 0.840,* P* < 0.001). Absence of AAC had a sensitivity, specificity, positive prognostic value, negative prognostic value, and accuracy of 46.7%, 95.9%, 94.1%, 56.4%, and 67.3%, respectively, for SS ≤22. AAC grades ≥2 had a sensitivity of 66.3%, specificity of 89.2%, positive prognostic value of 81.5%, negative prognostic value of 78.6%, and accuracy of 79.6% for the correct identification of SS >22.

**Conclusions:**

The extent of AAC detectable on chest X-rays might provide valuable information in predicting CAD severity in ACS patients.

## 1. Introduction

Patients with acute coronary syndrome (ACS) have a very high risk of cardiovascular (CV) morbidity and mortality, such that many of these patients undergo primary or elective coronary revascularization in combination with antithrombotic and lipid-lowering therapies to reduce elevated CV risk [1]. Among ACS patients managed with an early invasive strategy, baseline angiographic markers of disease burden, calcification, and lesion severity could provide important added independent predictive value for 30-day and 1-year ischemic outcomes [2]. The SYNergy between percutaneous coronary intervention with TAXus and cardiac surgery (SYNTAX) score (SS) is a valuable tool that can quantitatively estimate the severity of coronary lesions, and SS has been used to predict adverse CV events after coronary revascularization [3].

Arterial calcification has long been considered a complication of advanced atherosclerosis [4]. The aortic arch has been identified as the most vulnerable site for calcifications in the thoracic aorta [5]. Aortic arch calcification (AAC) is detectable on chest X-ray and accurately represents the magnitude of calcified change throughout the whole aorta [6]. Moreover, AAC can be detected readily and reproducibly using chest X-ray, which is a simple, inexpensive tool that is widely available for assessing hospitalized patients with chest pain. Several studies have reported that the presence of AAC could serve as an independent predictor of coronary artery calcium (CAC) presence, while the AAC extent was correlated with CAC scores [7, 8]. Furthermore, CAC has been demonstrated to provide incremental and independent power in predicting CAD severity [9, 10], suggesting that AAC might be a good predictor for CAD severity. Several recent studies have confirmed this assumption, indicating that AAC was associated with the severity of CAD, as evaluated by the number of diseased vessels or SS [11, 12]. However, these studies simply determined the presence of AAC without considering its extent and only included patients with non-ST-segment elevation ACS, excluding patients with ST-segment elevation myocardial infarction (MI).

In this cross-sectional study, we investigated the extent of AAC detectable in ACS patients from chest X-rays, determining if there was an association with the severity of CAD as evaluated by the SS.

## 2. Materials and Methods

### 2.1. Study Population

A total of 1,770 patients who underwent coronary angiography for ACS—and were treated with primary or elective coronary revascularization in our CV center between June 2016 and November 2017—were consecutively enrolled into a prospective registry. ACS was diagnosed according to the American College of Cardiology/American Heart Association guidelines [13, 14]. From the cohort, patients were excluded if they had prior coronary revascularization, were on chronic dialysis, or had been diagnosed with any known disease in the aorta, such as aortitis, aortic aneurysm, or dissection. Patients whose chest X-ray image quality was insufficient for interpretation were also excluded. Finally, 1,418 patients were included in the final analysis.

The present study was performed in accordance with the Helsinki Declaration of Human Rights and approved by the Medical Ethics Committee of Beijing Anzhen Hospital, Capital Medical University (Number: 2016034x). All patients provided written informed consent.

### 2.2. Measurements

Information on demographics, lifestyle, medical history, and daily medication use was collected with a detailed questionnaire. Weight, height, and blood pressure were measured following standardized procedures. All laboratory parameters were measured and analyzed immediately after collection from heparinized plasma samples at the central laboratory. Creatinine clearance (CrCl) was calculated using the Cockcroft and Gault formula [15]. Echocardiography was performed on admission and left ventricular ejection fraction was obtained using Simpson's method from apical 2- and 4-chamber views according to the established American Society of Echocardiography protocols [16].

### 2.3. Assessment of Aortic Arch Calcification

**Figure 1:**
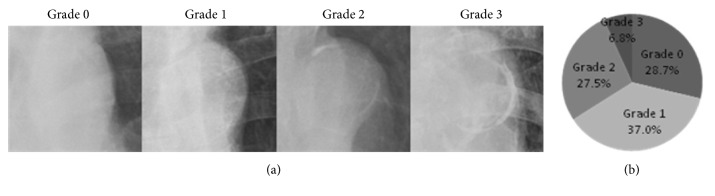
The aortic arch calcification (AAC) extent in four-point scale and distribution of AAC grades on a chest X -ray. (a) The extent of AAC on a chest X-ray was divided into four grades: grade 0, no visible calcification (panel A); grade 1, small spots of calcification or a single thin area of calcification of the aortic knob (panel B); grade 2, one or more areas of thick calcification (panel C); grade 3, circular calcification of the aortic knob (panel D). (b) Distribution of AAC grades on chest X-ray in all subjects.

All study patients received routine posterior-anterior chest X-rays (AXIOM Aristos MX, SIEMENS, Germany) or portable chest X-rays (MUX-200D, SHIMADZU, Japan) upon admission. AAC for each patient was assessed by two independent, experienced radiologists in a blinded fashion. If there was a dispute in interpretation, the opinion of another experienced radiologist was obtained and the final decision was made by consensus. The AAC extent was divided into four grades (0 to 3) (Figure 1(a)): grade 0, no visible calcification; grade 1, small spots of calcification or a single thin area of calcification of the aortic knob; grade 2, one or more areas of thick calcification; grade 3, circular calcification of the aortic knob [17].

### 2.4. SYNTAX Score

The SS was calculated based on each patient's coronary angiographic findings, using 11 angiographic factors that take into account lesion location and characteristics. All coronary lesions that had a diameter stenosis ≥50% in vessels ≥1.5 mm were scored in accordance with the SS algorithm as previously described [18]. The SS was defined as low (≤22), intermediate (23–32), and high (≥33). Intermediate and high SSs are associated with more complex CAD and represent a bigger therapeutic challenge, as well as a potentially worse prognosis [19]. Two experienced interventional cardiologists in an independent angiographic core laboratory—who were unaware of the patients' chest X-ray findings and clinical and laboratory parameters—independently performed angiographic visual analysis for calculating the SS. When disagreement occurred, the opinion of a third observer was obtained and the final decision was made by consensus.

Based on the results from the original trial [19], the patients were stratified into two groups: SS ≤22 (low SS group) and SS >22 (intermediate-to-high SS group).

### 2.5. Statistical Analysis

Continuous variables were expressed as the mean ± standard deviation if consistent with a normal distribution; otherwise, they were reported as the median (0.25–0.75 percentiles). Categorical variables were presented as numbers (percentages). Differences in two continuous variables were analyzed by the Student's t-test or the Mann-Whitney U test. Differences in categorical variables were tested using the chi-squared test or Fisher's exact test, as appropriate. SSs among the four AAC grades were compared using analysis of variance (F test) and polynomial contrast tests. Spearman's rank correlation coefficient (*ρ*) was calculated to evaluate the strength and direction of relationship between variables. Multivariate logistic regression analysis was performed to evaluate the independent contribution of the AAC extent to the risk of the intermediate-to-high SS. Effects of all variables on SS were calculated using univariate logistic regression analysis for each variable. Variables with a univariate significance level of ≤0.15 were entered into the multivariate logistic regression model. The linearity assumption between continuous variables and the logit in this model was assessed using the Box-Tidwell test. Fibrinogen and CrCl were found to be not linear as continuous variables with the logit and were therefore transformed into ordinal variables according to the quartiles of fibrinogen and generally accepted definition of chronic kidney disease (CKD) stages [20], respectively. All potential explanatory variables included in the multivariable analyses were subjected to collinearity analysis with a correlation matrix. The Hosmer–Lemeshow goodness-of-fit test was used to assess the fit of the logistic model. Odds ratios (ORs) and their 95% confidence intervals (CIs) were calculated. Receiver operating characteristic curve analysis was conducted to assess the discriminative performance of the AAC extent for the intermediate-to-high SS. The area under curve (AUC) that corresponded to 95% CIs was calculated. The predictive ability of the AAC extent for SS was evaluated. Sensitivity, specificity, positive predictive value (PPV), negative predictive value (NPV), and accuracy of the test were calculated. All statistical tests were two-tailed, and a* P* value <0.05 was considered to be statistically significant. Statistical analysis was performed using the IBM SPSS software (IBM SPSS Statistics for Windows, Version 24.0. Armonk, NY: IBM Corp.).

## 3. Results

A total of 1,418 patients were included in the present study. The study patients had a mean age of 59 ± 10 years and 24.3% (n = 344) of them were female. The mean SS value of the study population was 22 ± 11. The intraclass correlation efficient was 0.86 (*P* < 0.001) for the two interventional cardiologists who independently calculated SS. Within the cohort, 597 patients were allocated to the intermediate-to-high SS group, while 821 were allocated to the low SS group. The baseline characteristics, including the AAC extent, for all the patients based on SS are shown in Table 1. Patients with intermediate-to-high SS were older and had higher incidence of diabetes, prior MI, and peripheral vascular diseases than patients with low SS. Although there were no differences in hypertension between the two groups, patients in the intermediate-to-high SS group had a lower level of diastolic blood pressure. More patients developed acute MI in the intermediate-to-high SS group than in the low SS group.

**Figure 2:**
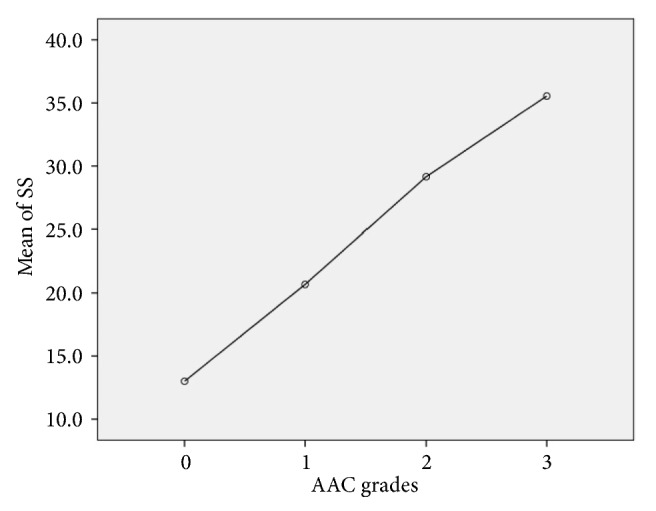
Distribution of SS across the AAC grades. Polynomial testing revealed a linear trend between SS and AAC grades (*P *< 0.001). There was a significant difference in SS among all four AAC grades: AAC grade 0 versus grade 1 (P < 0.001), 2 (P < 0.001), or 3 (P < 0.001); grade 1 versus grade 2 (P < 0.001) or 3 (P < 0.001); and grade 2 versus grade 3 (P < 0.001). Hollow circles indicate mean values. SS: SYNTAX score; AAC: aortic arch calcification.

Of all patients, 407 (28.7%), 524 (37.0%), 390 (27.5%), and 97 (6.8%) had AAC grades of 0, 1, 2, and 3, respectively (Figure 1(b)). Good interobserver agreement of the AAC extent was noted, with weighted *κ* statistics of 0.81 (95% CI 0.73–0.89). Mean SSs were 13 ± 7, 21 ± 9, 29 ± 9, and 36 ± 13 in patients with AAC grades 0, 1, 2, and 3, respectively (*P* <0.001). There was a linear trend between the four AAC grades and SSs (*P* < 0.001), and there was a significant difference in SSs among all four AAC grades: AAC grade 0 versus grade 1 (*P* < 0.001), 2 (*P* < 0.001), or 3 (*P *< 0.001); grade 1 versus grade 2 (*P* < 0.001) or 3 (*P* < 0.001); and grade 2 versus grade 3 (*P *< 0.001). The distribution of mean SS across the four AAC grades is shown in Figure 2. The AAC extent was significantly and positively correlated with SS (Rs = 0.639,* P* < 0.001) and heavy coronary calcification (Rs = 0.475,* P* < 0.001) (Table 2).

In the univariate analysis, compared to AAC grade 0, AAC grade 1 (OR 8.00, 95% CI 5.10–12.56), AAC grade 2 (OR 65.51, 95% CI 40.43–106.16), and AAC grade 3 (OR 98.21, 95% CI 47.96–201.08) were all the predictors of SS >22. In the multivariate logistic regression analysis, compared to AAC grade 0, ORs of AAC grades 1, 2, and 3 in predicting SS >22 were 12.95 (95% CI 7.85–21.36), 191.76 (95% CI 103.17–356.43), and 527.81 (95% CI 198.24–1405.28), respectively (Table 3).

**Figure 3:**
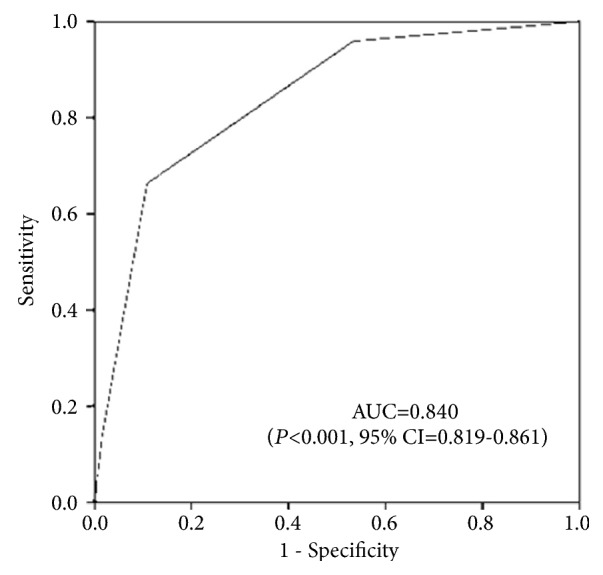
Receiver operating characteristic curve for AAC grades and SS >22. AUC: the area under curve; CI: confidence interval.

Receiver operating characteristic curve analysis yielded a strong predictive ability of the AAC extent for SS >22 (Figure 3, AUC = 0.840, 95% CI 0.819–0.861,* P *< 0.001).

Absence of AAC had a sensitivity, specificity, PPV, NPV, and accuracy of 46.7%, 95.9%, 94.1%, 56.4%, and 67.3%, respectively, for SS ≤22 (Table 4). An AAC grade of ≥2 predicted SS >22 with a sensitivity, specificity, PPV, NPV, and accuracy of 66.3%, 89.2%, 81.5%, 78.6%, and 79.6%, respectively (Table 4). An AAC grade of 3 had a sensitivity, specificity, PPV, NPV, and accuracy of 13.5%, 98.4%, 86.0%, 61.3%, and 62.9%, respectively, for SS >22 (Table 4). An AAC grade of 3 had a sensitivity of 24.0%, specificity of 97.1%, PPV of 63.4%, NPV of 85.8%, and accuracy of 83.4% for predicting a SS ≥33 (Table 4).

## 4. Discussion

To our knowledge, this is the first study to demonstrate a significant correlation of the extent of AAC detectable on a chest X-ray with the severity of CAD as evaluated by the SS in ACS patients. The predictive ability of absence of AAC was high for SS ≤22, while AAC grades 2 and 3 strongly predicted SS >22.

The SS is widely accepted as a CAD severity marker and its prognostic value has been demonstrated in a variety of different clinical scenarios, including ACS [21–23]. Although SS has remarkable clinical significance, it cannot be calculated quickly and easily, especially for clinicians who are not experienced in coronary angiography and intervention.

AAC, as a part of thoracic aortic calcification, has been established as a surrogate marker of atherosclerosis, better reflecting the total burden of atherosclerosis in one individual [24]. Previously, many studies have demonstrated that AAC is associated with similar CV risk factors as coronary atherosclerosis. In the Framingham study, AAC presence was associated with systolic and diastolic blood pressure, while also having a borderline association with total serum cholesterol [25]. In the Reykjavik study, AAC presence was independently associated with age, nonfasting plasma glucose, drug treatment for diabetes, blood pressure, use of antihypertensive agents, the amount of smoking, and serum cholesterol levels [26]. Iribarren et al. found that AAC was independently correlated with older age, current smoking, hypertension, and elevated total cholesterol levels [27]. Yamada et al. revealed that CKD in combination with diabetes—as well as hypertension in combination with CKD—strongly affected the risk of arterial calcification, especially at the aortic arch [28]. Symeonidis et al. categorized the AAC extent into four grades according to the amount, profile, and area of calcium on the aortic arch, revealing that the extent of AAC was significantly associated with older age, diabetes, hypertension, and dyslipidemia [17]. Similarly, Hashimoto et al. demonstrated that diabetes and renal dysfunction were significantly associated with increasing AAC grade [6]. The present study is consistent with these previous reports, finding a significant correlation between increasing AAC grade and traditional CV risk factors, such as older age, hypertension, diabetes, and CKD. Simultaneously, this study also found that patients with a higher AAC grade had a higher frequency of CV risk factors. In fact, there was heavier atherosclerotic plaque burden in CHD patients [29], which was concomitant with increased CV risk factors. Moreover, a number of angiographic and postmortem studies strongly support the notion that plaque burden severity significantly correlates with plaque instability and rupture [30].

In our study, we found a significant association between AAC and severe coronary calcification as defined by the SS algorithm. Several previous studies strongly and consistently support a significant, positive association between AAC on chest X-rays and CAC that is detected by computed tomography (CT) [7, 8]. Bannas et al. evaluated the association between AAC on chest X-rays and CAC scores that were determined by CT, where the AAC extent was divided into the same four grades (0-3) as the present study. Among 128 patients, the AAC extent was positively associated with CAC scores, while a cut-off between AAC grades 0–2 and 3 had a sensitivity of 38.6%, specificity of 96.4%, PPV of 85.0%, NPV of 75.0%, and accuracy of 76.6% for correctly identifying high‐risk CAC scores (> 400 Au). Adar et al. also investigated the association between AAC on chest X-rays and CAC that was detected by CT, where AAC was graded according to the same algorithm as this study. Among 248 patients, AAC was a strong and independent predictor of CAC, and an AAC grade of ≥2 (sensitivity 68%, specificity 98%, PPV 75%, NPV 97%, and accuracy 95%) or 3 (sensitivity 45%, specificity 99%, PPV 83%, NPV 95%, and accuracy 94%) had strong predictive power for high‐risk CAC scores (≥ 400 Au), while absence of AAC (sensitivity 90%, specificity 84%, PPV 96%, NPV69%, and accuracy 89%) had strong predictive performance for low‐risk CAC scores (< 100 Au). A recent genetic association study demonstrated that one specific single-nucleotide polymorphism (rs2026458) that was associated with CAC increased the risk of calcification in the aortic arch (*β* = 0.32, 95% CI 0.10–0.54,* P* = 0.004), demonstrating an internal correlation of CAC with AAC at the molecular level [31]. Previous studies have indicated that CAC detected by CT is associated with the presence and amount of coronary atherosclerosis, including the amount of noncalcified plaque, while other studies have revealed that CAC could provide incremental and independent power in predicting CAD severity [8, 9]. These associations may suggest a correlation of the AAC extent with CAD severity.

Currently, there is limited data regarding the association between AAC and CAD severity in ACS patients [11, 12]. Yun et al. evaluated the association between AAC and CAD severity in patients with unstable angina, where, among 178 consecutive patients, they demonstrated that AAC prevalence increased in proportion to CAD severity. However, this study did not consider the AAC extent and only used the number of diseased vessels to represent CAD severity. Korkmaz et al. evaluated the association between AAC and the severity of CAD as evaluated by SS in non-ST-segment elevation ACS patients, where, among 135 consecutive patients, AAC (95% CI 1.7–6.9,* P* = 0.002) was an independent determinant of SS in the linear regression analysis. However, this study did not consider the AAC extent and only had a small sample size.

At present, no data are available on the use of the AAC extent for the prediction of SS. In the present study, we found that an AAC grade of ≥2 (sensitivity 66.3%, specificity 89.2%, PPV 81.5%, NPV 78.6%, and accuracy 79.6%) or 3 (sensitivity 13.5%, specificity 98.4%, PPV 86.0%, NPV 61.3%, and accuracy 62.9%) had a high predictive power for SS >22. Moreover, we found a strong predictive performance of absence of AAC for SS ≤22 (sensitivity 46.7% specificity 95.9%, PPV, 94.1%, NPV 56.4%, and accuracy 67.3%). The extremely high specificity in our study population seems to advocate the AAC extent for the screening of SS ≤22 or >22 patients with ACS, where a high specificity is desired, even with a trade-off for sensitivity.

There are several limitations to this study. First, using chest X-rays to assess the AAC extent is not a quantitative method and the true calcium deposition in the aortic arch could have been underestimated. Second, positional change on the chest X-ray could potentially alter the appearance of AAC and influence the measured value of AAC thickness. Third, the calcification detectable on a chest X-ray is a composite of both intimal and medial calcifications, which are two pathophysiologically separate processes [32]. However, a chest X-ray does allow for accurate distinction between intimal and medial calcifications. The differences in the proportion of intimal and medial calcifications in the aortic arch may result in different relations with coronary atherosclerotic lesions. Finally, although this study demonstrated a close relationship between the extent of AAC detectable on a chest X-ray and the severity of CAD as evaluated by SS in ACS patients, there was a lack of prognostic information of AAC for future adverse CV events.

## 5. Conclusions

Our study demonstrates that the AAC extent is positively associated with the severity of CAD as evaluated by SS in ACS patients. Our data strongly suggest that the AAC extent, as assessed by a simplified approach by chest X-ray, might provide complementary and valuable information in predicting CAD severity for ACS patients.

## Figures and Tables

**Table 1:** Baseline characteristics of all patients and patients with low SS (SS ≤22) and intermediate-to-high SS (SS >22).

Variable	All Patients	Low SS	Intermediate-to-high SS	*P *value
	N = 1,418	N = 821	N = 597	
*Female gender, n (*%)	344 (24.3)	203 (24.7)	141 (23.6)	0.503
*Age (years)*	59±10	58±10	61±11	<0.001
*Height (m)*	1.69 (1.63–1.73)	1.69 (1.63–1.73)	1.68 (1.62–1.73)	0.269
*Weight (kg)*	73±12	73±12	72±12	0.427
*BMI (kg/m* ^*2*^)	25.7±3.3	25.7±3.3	25.7±3.3	0.834
*Family history of CHD, n (*%)	428 (30.2)	244 (29.7)	184 (30.8)	0.766
*Arterial hypertension, n (*%)	904 (63.8)	513 (62.5)	391 (65.5)	0.248
*Diabetes, n (*%)	542 (38.2)	260 (31.7)	282 (47.2)	<0.001
*Dyslipidemia, n (*%)	1120 (79.0)	636 (77.5)	484 (81.1)	0.088
*History of MI, n (*%)	148 (10.4)	56 (6.8)	92 (15.4)	<0.001
*History of CA, n (*%)	90 (6.3)	44 (5.4)	46 (7.7)	0.066
*Known PVD, n (*%)	122 (8.6)	31 (3.8)	91 (15.2)	<0.001
*COPD, n (%)*	18 (1.3)	12 (1.5)	6 (1.0)	0.461
*Smoking*				0.012
Never smokers, n (%)	592 (41.7)	339 (41.3)	253 (42.4)	
Former smokers, n (%)	158 (11.1)	76 (9.3)	82 (13.7)	
Current smokers, n (%)	668 (47.1)	406 (49.5)	262 (43.9)	
*ACS types*				0.002
UA, n (%)	980 (69.1)	595 (72.5)	385 (64.5)	
NSTEMI, n (%)	204 (14.4)	100 (12.2)	104 (17.4)	
STEMI, n (%)	234 (16.5)	126 (15.3)	108 (18.1)	
*Medical measurements (on admission)*				
SBP (mmHg)	130±17	130±17	130±17	0.999
DBP (mmHg)	76±11	77±11	75±11	0.021
*Laboratory measurements (on admission)*				
CK-MB (ng/ml)	1.3 (0.9–1.9)	1.2 (0.9–1.9)	1.4 (0.9–2.0)	0.015
cTnI (ng/ml)	0 (0–0.02)	0 (0–0.01)	0.01 (0–0.04)	<0.001
BNP (pg/ml)	36 (22–87)	33 (20–67)	43 (23–108)	<0.001
WBC count (10^9^/L)	6.43 (5.38–7.67)	6.41 (5.42–7.68)	6.47 (5.33–7.67)	0.651
Fibrinogen (g/L)	3.22 (2.80–3.68)	3.16 (2.77–3.55)	3.31 (2.92–3.82)	<0.001
CRP (mg/L)	1.37 (0.55–4.10)	1.23 (0.51–3.43)	1.67 (0.63–5.64)	<0.001
*Laboratory measurements (fasting state)*				
TC (mmol/L)	4.18±1.02	4.18±1.01	4.18±1.02	0.885
LDL-C (mmol/L)	2.49±0.83	2.46±0.83	2.53±0.83	0.121
HDL-C (mmol/L)	1.00 (0.87–1.17)	1.02 (0.87–1.18)	0.97 (0.86–1.16)	0.003
TG (mmol/L)	1.43 (1.03–2.10)	1.51 (1.05–2.13)	1.37 (1.00–1.98)	0.012
FPG (mmol/L)	5.13 (5.66–6.99)	5.49 (5.05–6.64)	5.99 (5.26–7.45)	<0.001
HbA1c (%)	5.9 (5.5–6.9)	5.8 (5.5–6.6)	6.2 (5.6–7.4)	<0.001
CrCl (ml/min)	102±31	104±32	98±28	<0.001
*LVEF (*%)	65 (60–68)	65 (60–69)	64 (60–68)	<0.001
*Medications use (before admission)*				
Antiplatelet drugs, n (%)	982 (69.3)	559 (68.1)	423 (70.9)	0.322
HMG-CoA inhibitors, n (%)	958 (67.6)	545 (66.4)	413 (69.2)	0.326
ACE inhibitors/ARBs, n (%)	350 (24.7)	198 (24.1)	152 (25.5)	0.587
Beta-blockers, n (%)	517 (36.5)	270 (32.9)	247 (41.4)	0.002
CCBs, n (%)	467 (32.9)	248 (30.2)	219 (36.7)	0.008
Insulin, n (%)	202 (14.2)	76 (9.3)	126 (21.1)	<0.001
Sulfonyl Urea, n (%)	122 (8.6)	57 (6.9)	65 (10.9)	0.013
Biguanides, n (%)	136 (9.6)	72 (8.8)	64 (10.7)	0.276
Alpha-glucosidase inhibitors, n (%)	98 (6.9)	44 (5.4)	54 (9.0)	0.006
*Angiographic characteristics*				
Left-main disease, n (%)	116 (8.2)	18 (2.2)	98 (16.4)	<0.001
Three-vessel disease, n (%)	786 (55.4)	294 (35.8)	492 (82.4)	<0.001
Proximal LAD stenosis, n (%)	780 (55.0)	321 (39.1)	459 (76.9)	<0.001
Trifurcation or bifurcation lesions, n (%)	1094 (77.2)	554 (67.5)	540 (90.5)	<0.001
Total occlusions, n (%)	390 (27.5)	130 (15.8)	260 (43.6)	<0.001
Heavy calcification lesions, n (%)	432 (30.5)	116 (14.1)	316 (52.9)	<0.001
Lesions length >20mm, n (%)	750 (52.9)	323 (39.3)	427 (71.5)	<0.001
*AAC grades*				<0.001
Grade 0, n (%)	407 (28.7)	383 (46.7)	24 (4.0)	
Grade 1, n (%)	524 (37.0)	349 (42.5)	175 (29.3)	
Grade 2, n (%)	390 (27.5)	76 (9.3)	314 (52.6)	
Grade 3, n (%)	97 (6.8)	13 (1.6)	84 (14.1)	

SS: SYNTAX score; BMI: body mass index; CHD: coronary heart disease; CA: cerebrovascular accident; PVD: peripheral vascular disease; COPD: chronic obstructive pulmonary disease; MI: myocardial infarction; UA: unstable angina; NSTEMI: non-ST-segment elevation myocardial infarction; STEMI: ST-segment elevation myocardial infarction; SBP: systolic blood pressure; DBP: diastolic blood pressure; CK-MB: creatine kinase isoenzyme MB; cTnI: cardiac troponin I; BNP: brain natriuretic peptide; WBC: white blood cell; CRP: C-reactive protein; TC: total cholesterol; LDL-C: low-density lipoprotein-cholesterol; HDL-C: high-density lipoprotein-cholesterol; TG: triglyceride; FPG: fasting plasma glucose; HbA1c: glycated haemoglobin A1c; CrCl: creatinine clearance; LVEF: left ventricular ejection fraction; ACE: angiotensin converting enzyme; ARBs: angiotensin II receptor blockers; CCBs: calcium channel blockers; LAD: left anterior descending; AAC: aortic arch calcification.

**Table 2:** Correlation analysis between AAC, heavy coronary calcification, and SS.

	AAC grades and SS	Coronary	Coronary calcification
		calcification and SS	and AAC grades
*ρ*	0.639	0.459	0.475
*P* value	<0.001	<0.001	<0.001

Abbreviations: see Table 1.

**Table 3:** The predictive value of AAC grades for intermediate–high SS.

Variable	Univariate	*P* value	Multivariate^*∗*^	*P *value
	OR, 95% CI		OR, 95% CI	
AAC grade 0	Reference		Reference	
AAC grade 1	8.00, 5.10–12.56	<0.001	12.95, 7.85–21.36	<0.001
AAC grade 2	65.51, 40.43–106.16	<0.001	191.76, 103.17–356.43	<0.001
AAC grade 3	98.21, 47.96–201.08	<0.001	527.81, 198.24–1405.28	<0.001

^*∗*^Other variables included in multivariable analysis were age, diabetes, dyslipidemia, history of MI, history of CA, known PVD, smoking, ACS types, DBP, CK-MB, cTnI, fibrinogen categories, CRP, LDL-C, HDL-C, TG, FPG, HbA1c, CKD stages, LVEF, beta-blockers, CCBs, insulin, sulfonyl urea, and alpha-glucosidase inhibitors.

OR indicates odds ratio; for other abbreviations, see Table 1.

**Table 4:** Predictive ability of AAC grades for SS.

Predictor (AAC grades)	Outcome (SS)	Sensitivity (%)	Specificity (%)	PPV (%)	NPV (%)	Accuracy (%)
Grade =0	*≤22*	46.7	95.9	94.1	56.4	67.3
Grade ≥2	*>22*	66.3	89.2	81.5	78.6	79.6
Grade =3	*>22*	13.5	98.4	86.0	61.3	62.9
Grade =3	*≥33*	24.0	97.1	63.4	85.8	84.3

AAC: aortic arch calcification; SS: SYNTAX score; PPV: positive predictive value; NPV: negative predictive value.

## Data Availability

The data used to support the findings of this study are available from the corresponding author upon request.
